# National trends in the prevalence of diabetic retinopathy among Thai patients with type 2 diabetes and its associated factors from 2014 to 2018

**DOI:** 10.1371/journal.pone.0245801

**Published:** 2021-01-22

**Authors:** Nathakamol Euswas, Napat Phonnopparat, Kantapat Morasert, Pongpisut Thakhampaeng, Apisit Kaewsanit, Mathirut Mungthin, Ram Rangsin, Boonsub Sakboonyarat

**Affiliations:** 1 Phramongkutklao College of Medicine, Bangkok, Thailand; 2 Department of Military and Community Medicine, Phramongkutklao College of Medicine, Bangkok, Thailand; 3 Department of Anatomy, Phramongkutklao College of Medicine, Bangkok, Thailand; 4 Department of Pharmacology, Phramongkutklao College of Medicine, Bangkok, Thailand; Virginia Commonwealth University, UNITED STATES

## Abstract

**Introduction:**

Diabetic retinopathy (DR) is one of the most common and serious ocular complications in both developed and developing countries. To date, epidemiological data of DR and their associated factors in Southeast Asian countries especially in Thailand are scarce. We aimed to use the information from the Thailand Diabetes Mellitus/Hypertension (DM/HT) study to determine trends in DR prevalence and also risk factors among Thai patients with type 2 diabetes (T2D).

**Methods:**

A series of cross-sectional surveys of clinical outcomes was conducted annually in 2014, 2015 and 2018 among patients with T2D aged >18 years receiving medical care for at least 12 months. A stratified single stage cluster sampling method that was proportional to the size sampling technique was used to select a nationally and provincially representative sample of patients with T2D in Thailand. A standardized case report form was used to obtain the required information from medical records.

**Results:**

A total of 104,472 Thai patients with T2D were included in the study from 2014 to 2018. The dominant proportion of participants, 70,756 (67.7%), were females. The overall prevalence of DR declined from 6.9% in 2014 to 6.3% in 2015 and 5.0% in 2018 (*p* for trend <0.001). The independent associated factors with DR included survey year, greater duration of DM, geographic region, hospital level, social security scheme, dyslipidemia, insulin therapy, high HbA1c level and elevated pulse pressure.

**Conclusion:**

We reported a decreasing in trend in the prevalence of DR among Thai patients with T2D over one half decade. Patients with T2D should be encouraged to control their underlying diseases and engage in other effective interventions. When these modifiable risk factors can be inhibited, DR and other cardiovascular complications will be alleviated.

## Introduction

Globally, adults with diabetes mellitus (DM) totaled 381 million in 2013 and were projected to 463 million in 2019 [1, 2]. In Thailand, the prevalence of type 2 diabetes (T2D) among adults rose from 2.3% in 1991 [3] to 8.0% in 2015 [4, 5]. Diabetic retinopathy (DR) is one of the most common and serious ocular complications in both developed and developing countries [6]. The estimated prevalence of DR among patients with T2D was 25.2, 22.2, 40.0 and 43.0% worldwide [7], in Italy [8], the US [9] and China [10], respectively. The presence of DR reflects microcirculatory diseases in vital organs and also relates to a higher risk for systemic vascular complications including stroke and ischemic heart disease [11]. Additionally, patients with DM and DR have a significantly lower quality of life when compared with those without DR in any aspect including effect on general health, general vision and mental health. Moreover, decreased quality of life was associated with the duration of retinopathy and severity of retinopathy [12]. Many risk factors of DR are associated with complications that have been studied worldwide [6,13–15]. However, epidemiological data for DR in Southeast Asian countries especially Thailand are scarce. A limited information is available of the distribution of DR prevalence by geographic region, hospital level, or health coverage scheme in Thailand. Thus, we aimed to use the information from the Thailand Diabetes Mellitus/Hypertension (DM/HT) study from 2014 to 2018 to determine trends in DR prevalence and risk factors among Thai patients with T2D. Additionally, we would like to explore the association between the demographic data of participants including geographic region, hospital level, health coverage scheme and DR prevalence. Our findings may be used to improve healthcare services access of patients with T2D and also determine effective public health interventions of diabetes care for Thai patients with T2D.

## Methods

### Study designs and subjects

A series of annual cross-sectional surveys was conducted in 2014, 2015 and 2018. The data were retrieved from the database: An Assessment on Quality of Care among Patients Diagnosed with Type 2 Diabetes and Hypertension Visiting the Ministry of Public Health (MoPH) and Bangkok Metropolitan Administration Hospitals in Thailand (Thailand DM/HT) after obtaining permission from the National Health Security Office (NHSO) and Medical Research Network of the Consortium of Thai Medical Schools (MedResNet). All hospitals under the MoPH at all levels, i.e., health promoting (subdistrict), community (district), general (provincial) and regional hospitals nationwide were invited to participate in the Thailand DM/HT study. A total of 1,098 MoPH hospitals were categorized as 28 regional hospitals, 80 general hospitals, 883 community hospitals and 107 health promoting hospitals. A stratified single stage cluster sampling method proportional to the size was used to select national and provincial representative samples of patients with T2D in Thailand. Inclusion criteria for this study comprised patients with T2D aged at least 18 years receiving medical treatment in an MoPH hospital, drawn from those sampling methods, during the previous 12 months. Any patient who had participated in a clinical trial was excluded. The participant populations totaled 33,268 in 2014, 32,616 in 2015 and 38,568 in 2018.

### Data collection

A standardized case report form (CRF), used to collect the data indicating care among patients with T2D from consecutive patient’s medical records, included demographic characteristics, status of DM complications and result of laboratory tests. The data from all hospitals were sent to the central data management unit of MedResNet. Collected data included demographics, weight, height, body mass index (BMI), smoking behavior, systolic blood pressure (SBP), diastolic blood pressure (DBP), fasting plasma glucose (FPG), hemoglobin A1c (HbA1c), low density lipoprotein cholesterol (LDL), insulin therapy, diagnosing DR and comorbidities including hypertension (HT), dyslipidemia (DLP) and gout. DM was defined by Diabetes Care, 2014 as FPG ≥126 mg/dl and confirmed by repeat testing at a second visit, or HbA1c ≥6.5% [16]. DR was determined according to claims using the International Classification of Diseases, Tenth Revision codes in E113 which appeared in the medical records [17]. Smoking was defined as those who currently smoked (within the last 12 months) and never smoked (patients who had never smoked, or who had smoked less than 100 cigarettes in their lifetime). Ex-smoker was defined by smoke-free for 12 months [18]. BMI was calculated as body weight in kilograms divided by height in meters squared [weight(kg)/height(m)^2^]. The pulse pressure (PP) was calculated as SBP level minus DBP level and categorized in three groups including 1^st^ quartile (Q_1_), 2^nd^ quartile(Q_2_) to 3^rd^ quartile (Q_3_) and 4^th^ quartile (Q4).

### Statistical analysis

Data was analyzed using StataCorp. 2015. *Stata Statistical Software*: *Release 14*, College Station, TX, USA: StataCorp LP. Demographic characteristics were determined using descriptive statistics. Categorical data were presented as number and percentage while continuous data were presented as mean and standard deviation (SD). Prevalence of DR was determined using descriptive statistics and reported as a percentage with 95% confidence interval (95% CI). *P* for trend was calculated using *chi*-square statistics for trends. The *chi*-square test was used to compare categorical data while continuous data were compared using Student’s *t*-test. Binary logistic regression analysis was used to determine the associated factors for DR, and the magnitude of association was presented as crude odds ratio (OR) with 95%CI. Multivariate analysis was performed using logistic regression analysis. Adjusted odds ratio (AOR) from multivariate analysis was presented with corresponding 95% CI, and statistical significance was set at *p*-value <0.05.

### Ethics consideration

The Thailand DM/HT study was approved by the Thai National Health Security Office institutional review board. The participants provided written consent in agreement with the WMA Declaration of Helsinki—Ethics principles for medical research involving human subjects. This study was reviewed and approved by the Royal Thai Army Medical Department Institutional Review Board (approval number R192h/62_Exp).

## Results

### Demographic characteristics

A total of 104,472 Thai patients with T2D were included in the study comprising 33,288 (31.9%) in 2014, 32,616 (31.2%) in 2015 and 38,568 (36.9%) in 2018. The dominant proportion of participants, 70,756 (67.7%), were females. The average age of participants was 61.1±11.0, 61.5±11.0 and 62.3±11.0 years while the average of duration of DM after diagnosis was 7.6±4.1, 7.7±4.9 and 7.8±5.2 years in 2014, 2015, and 2018, respectively. Demographic characteristics of the study participants by year are presented in Table 1.

**Table 1:** Demographic characteristics of participants (n = 104472).

Year	2014	2015	2018
Characteristics	n = 33288	n = 32616	n = 38568
	n (%)	n (%)	n (%)
**Sex**			
Male	10345 (31.1)	10603 (32.5)	12748 (33.1)
Female	22923 (68.9)	22013 (67.5)	25820 (66.9)
**Age (years)**			
18–30	68 (0.2)	63 (0.2)	68 (0.2)
30–39	796 (2.4)	714 (2.2)	718 (1.9)
40–49	4144 (12.5)	3683 (11.3)	3874 (10.0)
50–59	9675 (29.1)	9420 (28.9)	10645 (27.6)
60–69	10997 (33.1)	10965 (33.6)	13334 (34.6)
70–79	6078 (18.3)	6178 (18.9)	7501 (19.4)
≥80	1499 (4.5)	1593 (4.9)	2428 (6.3)
Mean±S.D.	61.1±11.0	61.5±11.0	62.3±11.0
**Geographic region**			
North	6680 (20.1)	6972 (21.4)	8920 (23.1)
Central	10252 (30.8)	11571 (35.5)	12505 (32.4)
Northeast	11783 (35.4)	9444 (29.0)	10610 (27.5)
South	4573 (13.7)	4629 (14.2)	6533 (16.9)
**Hospital level**			
Regional hospital (S/A)	2665 (8.0)	2919 (9.0)	2670 (6.9)
General hospital	5990 (18.0)	7838 (24.0)	7554 (19.6)
Community Hospital	24633 (74.0)	21859 (67.0)	26569 (68.9)
Health Promoting Hospital	n/a	n/a	1775 (4.6)
**Occupation**			
Agriculturist	13700 (41.2)	12305 (37.7)	14030 (36.4)
Retirement	9739 (29.3)	9869 (30.3)	12756 (33.1)
Employee	4894 (14.7)	5091 (15.6)	5946 (15.4)
Private business	1980 (5.9)	2437 (7.5)	2578 (6.7)
Government officer	1307 (3.9)	1538 (4.7)	1764 (4.6)
Others	1668 (5.0)	1376 (4.2)	1494 (3.9)
**Religion**			
Buddhist	29905 (95.8)	29977 (96.0)	35573 (94.4)
Islamic	1233 (4.0)	1212 (3.9)	2002 (5.3)
Christian	62 (0.2)	49 (0.2)	93 (0.2)
**Scheme**			
Universal healthcare coverage	26245 (79.0)	24905 (76.7)	30269 (78.6)
Civil servant medical benefit	5219 (15.7)	5716 (17.6)	6270 (16.3)
Social security	1327 (4.0)	1335 (4.1)	1523 (4.0)
Others	436 (1.3)	529 (1.6)	448 (1.1)
**Hypertension**	25379 (76.2)	25520 (78.2)	30113 (78.1)
**Dyslipidemia**	23059 (69.3)	23865 (73.2)	27178 (70.5)
**Gout**	1278 (3.8)	1328 (4.1)	2160 (5.6)
**BMI (kg/m**^**2**^**)**			
Mean±S.D.	25.5±4.6	25.7±4.6	25.7±4.8
**DM Duration (years)**			
Mean±S.D.	7.6±4.1	7.7±4.9	7.8±5.2
**FPG(mg/dl)**			
Mean±S.D.	153.7±55.5	153.9±55.8	153.5±54.3
**HbA1c (%)**			
Mean±S.D.	8.0±2.1	7.9±2	7.9±2

Regional hospital (S/A); regional hospital (standard/advanced) SD; standard deviation, BMI; Body mass index, DM; diabetes mellitus, FPG; fasting plasma glucose, kg/m^2^; kilogram/square meter, mg/dl; milligram/deciliter

#### Trends in the prevalence of DR among Thai patients with T2D

From 2014 to 2018, the overall prevalence of DR among Thai patients with T2D decreased significantly over 5 years. Table 2 illustrates the trends in the prevalence of DR by sex, age groups, geographic region, and hospital level. The overall prevalence of DR declined from 6.9% in 2014 to 6.3% in 2015 and 5.0% in 2018 (*p* for trend < 0.001). The DR prevalence among males continuously decreased from 6.5 to 6.1 and 5.0% in 2014, 2015 and 2018, respectively, (*p* for trend <0.001). Among females, a significant decreasing trend was found in DR prevalence from 7.1 to 6.4 and 5.1% in 2014, 2015 and 2018, respectively (*p* for trend <0.001). No difference was found in the prevalence of DR among patients with T2D in regional hospitals in 2014, 2015 and 2018 *(p*-value = 0.841), whereas, DR prevalence among patients with T2D in general hospitals and community hospitals tended to decline from 2014 to 2018.

**Table 2:** Trends in the prevalence of diabetic retinopathy (DR) among Thai patients with T2D, 2014–2018.

Characteristics	2014	2015	2018	*p* for trend
**Sex**				
Male	6.52 (6.04–6.99)	6.06 (5.61–6.52)	5.00 (4.63–5.38)	<0.001
Female	7.13 (6.80–7.46)	6.36 (6.04–6.68)	5.05 (4.79–5.32)	<0.001
**Age (years)**				
<40	5.90 (4.33–7.48)	5.41 (3.81–7.00)	4.83 (3.33–6.34)	0.364
40–49	7.02 (6.24–7.80)	6.00 (5.23–6.77)	4.78 (4.10–5.45)	<0.001
50–59	7.78 (7.25–8.31)	6.88 (6.37–7.39)	5.47 (5.04–5.90)	<0.001
≥60	6.54 (6.19–6.90)	6.04 (5.70–6.38)	4.89 (4.61–5.17)	<0.001
**Geographic region**				
North	6.87 (6.26–7.48)	6.11 (5.55–6.67)	5.53 (5.05–6.00)	0.001
Central	8.09 (7.56–8.61)	7.01 (6.54–7.47)	5.22 (4.83–5.61)	<0.001
Northeast	6.23 (5.79–6.67)	5.16 (4.71–5.60)	3.65 (3.29–4.00)	<0.001
South	6.30 (5.59–7.00)	6.89 (6.16–7.62)	6.28 (5.69–6.86)	0.864
**Hospital level**				
Regional hospital (S/A)	12.08 (10.84–13.32)	11.99 (10.81–13.17)	12.28 (11.04–13.53)	0.841
General hospital	11.34 (10.53–12.14)	9.24 (8.60–9.88)	7.55 (6.95–8.14)	<0.001
Community hospital	5.31 (5.03–5.59)	4.43 (4.16–4.71)	3.88 (3.64–4.11)	<0.001
Health promoting hospitals	n/a	n/a	0.85 (0.41–1.27)	n/a
**Total**	6.94 (6.67–7.21)	6.26 (6.00–6.53)	5.04 (4.82–5.26)	<0.001

Regional hospital (S/A); regional hospital (standard/advanced)

#### Associated factors of DR among Thai patients with T2D

Univariate logistic regression analyses were performed to determine factors associated with DR, as presented in Table 3. The independent associated factors with DR among Thai patients with T2D from 2014 to 2018 are illustrated in Table 4. After adjusting for potential confounders, factors associated with DR included survey year, greater duration of DM, geographic region, hospital level, Social Security Scheme (SSS), DLP comorbidity, insulin therapy, HbA1c level and PP.

**Table 3:** Univariable analysis for factors associated with diabetic retinopathy (DR) among Thai patients with T2D, 2014–2018.

Factors	DR	Non-DR	Crude	95%CI	*p*-value
	n(%)	n(%)	Odds Ratio		
**Year**					
2014	2310 (6.9)	30978 (93.1)	1.00		
2015	2043 (6.3)	30573 (93.7)	0.90	0.84–0.95	<0.001
2018	1943 (5.0)	36625 (95.0)	0.71	0.67–0.76	<0.001
**Sex**					
Male	1955 (5.8)	31741 (94.2)	1.00		
Female	4339 (6.1)	66417 (93.9)	1.06	1.00–1.12	0.036
**Age (years)**					
<40	131 (5.4)	2296 (94.6)	1.00		
40–49	697 (6.0)	11004 (94.0)	1.11	0.92–1.35	0.286
50–59	1983 (6.7)	27757 (93.3)	1.25	1.04–1.50	0.015
≥60	3485 (5.8)	57088 (94.2)	1.07	0.89–1.28	0.460
Mean±S.D.	61.0±10.4	61.7±11.0	0.99	0.99–0.99	<0.001
**DM Duration (years)**					
<10	3197 (4.6)	66453 (95.4)	1.00		
10–19	2395 (8.7)	25279 (91.4)	1.97	1.86–2.08	<0.001
≥20	343 (15.3)	1894 (84.7)	3.76	3.34–4.25	<0.001
**Geographic region**					
North	1378 (6.1)	21194 (93.9)	1.00		
Central	2293 (6.7)	32035 (93.3)	1.10	1.03–1.18	0.006
Northeast	1608 (5.1)	30229 (94.9)	0.82	0.76–0.88	<0.001
South	1017 (6.5)	14718 (93.5)	1.06	0.98–1.16	0.154
**Hospital level**					
Regional hospital (S/A)	1000 (12.1)	7254 (87.9)	1.00		
General hospital	1973 (9.2)	19409 (90.8)	0.74	0.68–0.80	<0.001
Community hospital	3308 (4.5)	69753 (95.5)	0.34	0.32–0.37	<0.001
Health promoting hospital	15 (0.8)	1760 (99.2)	0.06	0.04–0.10	<0.001
**Scheme**					
Universal healthcare coverage	4769 (5.9)	76650 (94.1)	1.00		
Civil servant medical benefit	1064 (6.2)	16141 (93.8)	1.06	0.99–1.13	0.099
Social security	355 (8.5)	3830 (91.5)	1.49	1.33–1.67	<0.001
Others	85 (6.0)	1328 (94.0)	1.02	0.82–1.28	0.802
**Smoking**					
Never	5206 (6.1)	80490 (93.9)	1.00		
Current	218 (5.2)	3960 (94.8)	0.85	0.74–0.98	0.023
Ex-smoker	615 (6.0)	9665 (94.0)	0.98	0.90–1.07	0.711
**Hypertension**					
No	1013 (4.3)	22447 (95.7)	1.00		
Yes	5283 (6.5)	75729 (93.5)	1.55	1.44–1.67	<0.001
**Dyslipidemia**					
No	1458 (4.8)	28912 (95.2)	1.00		
Yes	4838 (6.5)	69264 (93.5)	1.39	1.30–1.47	<0.001
**Gout**					
No	6017 (6.0)	93689 (94.0)	1.00		
Yes	279 (5.9)	4487 (94.1)	0.97	0.86–1.10	0.608
**Insulin therapy**					
No	3691 (4.5)	77556 (95.5)	1.00		
Yes	2605 (11.2)	20620 (88.8)	2.66	2.56–2.80	<0.001
**HbA1c (%)**					
<7.0	1394 (4.7)	28229 (95.3)	1.00		
7.0–7.9	1155 (6.0)	18008 (94.0)	1.30	1.20–1.41	<0.001
8.0–8.9	860 (6.8)	11877 (93.2)	1.47	1.34–1.60	<0.001
≥9.0	1801 (8.7)	18987 (91.3)	1.92	1.79–2.07	<0.001
Mean±S.D.	8.5±2.1	7.9±2.0	1.12	1.11–1.14	<0.001
**LDL (mg/dl)**					
<70	731 (5.6)	12316 (94.4)	1.00		
≥70	4851 (6.0)	76090 (94.0)	1.07	0.99–1.16	0.080
Mean±S.D.	109.2±40.4	107.4±37.7	1.01	1.00–1.01	0.001
**BMI (kg/m**^**2**^**)**					
<18.5	219 (5.6)	3717 (94.4)	1.00		
18.5–22.9	1620 (6.2)	24488 (93.8)	1.12	0.97–1.30	0.118
23.0–24.9	1205 (6.0)	19001 (94.0)	1.08	0.93–1.25	0.330
25.0–29.9	2085 (5.8)	33870 (94.2)	1.05	0.91–1.21	0.549
≥30.0	970 (6.2)	14767 (93.8)	1.12	0.96–1.30	0.158
Mean±S.D.	25.6±4.7	25.6±4.7	1.00	0.99–1.01	0.848
**SBP (mmHg)**					
<140	4119 (5.5)	70270 (94.5)	1.00		
≥140	2169 (7.3)	27746 (92.7)	1.33	1.26–1.41	<0.001
Mean±S.D.	134.0±17.0	131.5±15.8	1.01	1.01–1.01	<0.001
**DBP (mmHg)**					
<90	5774 (6.1)	89457 (93.9)	1.00		
≥90	514 (5.7)	8559 (94.3)	0.93	0.85–1.02	0.128
Mean±S.D.	73.6±10.5	74.5±10.2	0.99	0.99–0.99	<0.001
**Pulse pressure (mmHg)**					
<56 (<Q2)	2516 (5.0)	47980 (95.0)	1.00		
56–66 (Q2-Q3)	1757 (6.1)	27150 (93.9)	1.23	1.16–1.31	<0.001
>66 (>Q3)	2015 (8.1)	22886 (91.9)	1.68	1.58–1.78	<0.001
Mean±S.D.	60.3±15.5	57.0±14.4	1.02	1.01–1.02	<0.001

Regional hospital (S/A); regional hospital (standard/advanced) SD; standard deviation, LDL; low-density lipoprotein cholesterol, BMI; Body mass index, SBP; systolic blood pressure, DBP; diastolic blood pressure, mmHg; millimeters of mercury; kg/m^2^; kilogram/square meter, mg/dl; milligram/deciliter, 95% CI; 95% confidence interval

**Table 4:** Multivariate analysis for factors associated with diabetic retinopathy (DR) among Thai patients with T2D, 2014–2018.

Factors	Adjusted Odds Ratio	95%CI	*p*-value
**Year**			
2014	1.00		
2015	0.77	0.72–0.83	<0.001
2018	0.66	0.62–0.71	<0.001
**Sex**			
Male	1.00		
Female	0.98	0.92–1.05	0.558
**Age (years)**			
<40	1.00		
40–49	1.20	0.96–1.50	0.136
50–59	1.26	1.02–1.56	0.029
≥60	0.95	0.77–1.18	0.656
**DM Duration (years)**			
<10	1.00		
10–19	1.66	1.56–1.77	<0.001
≥20	2.88	2.50–3.32	<0.001
**Geographic regions**			
North	1.00		
Central	1.18	1.10–1.28	<0.001
Northeast	0.90	0.82–0.98	0.017
South	1.17	1.06–1.28	0.002
**Hospital level**			
Regional hospital (Standard/Advanced) 1.00			
General hospital	0.79	0.72–0.87	<0.001
Community hospital	0.37	0.34–0.40	<0.001
Health promoting hospital	0.13	0.07–0.23	<0.001
**Scheme**			
Universal healthcare coverage	1.00		
Civil servant medical benefit	0.90	0.83–0.98	0.017
Social security	1.17	1.02–1.33	0.026
Others	0.89	0.69–1.15	0.378
**DLP**			
No	1.00		
Yes	1.18	1.10–1.27	<0.001
**Insulin therapy**			
No	1.00		
Yes	2.19	2.05–2.34	<0.001
**HbA1c level (%)**			
<7.0	1.00		
7.0–7.9	1.16	1.07–1.27	0.001
8.0–8.9	1.23	1.12–1.35	<0.001
≥9.0	1.45	1.34–1.58	<0.001
**Pulse pressure (mmHg)**			
1^st^ Quartile (<56)	1.00		
2^nd^ -3^rd^ Quartile (56–66)	1.25	1.17–1.35	<0.001
4^th^ Quartile (>66)	1.64	1.52–1.76	<0.001

DM; diabetes mellitus, Regional hospital (S/A); regional hospital (standard/advanced) LDL; low-density lipoprotein cholesterol, 95% CI; 95% confidence interval

## Discussion

To our knowledge, this is the first report using the largest epidemiological study in Southeast Asia, focusing on DR and its associated factors among Thai patients with T2D. These results revealed the essential evidence of decreasing trends in the prevalence of DR among Thai patients with T2D from 2014 to 2018. The overall prevalence of DR among Thai patients with T2D was 5.0 to 6.9%. Compared with the prevalence of DR among patients with T2D globally and in other countries including Italy, the US and China, the prevalence of DR in Thailand was relatively low [7–10]. Additionally, the Thailand diabetes registry project 2003, conducted in 11 tertiary hospitals, reported a DR prevalence of approximately 31.4% [19]. Our study reported that since 2014 the overall prevalence of DR among patients with T2D significantly dropped over one half decade. This finding may be explained by improved diabetic care due to Thai national health policy. Firstly, since 2002, the universal healthcare coverage was established and by 2013 it covered 99.8% of the Thai population [20]. Therefore, Thai patients with T2D had more opportunity to access medical care. Moreover, the Thai clinical practice guidelines (CPG) for diabetes was established since 2011 by the Diabetes Association of Thailand and updated by following the American Diabetes Association’s Standards of Medical Care in Diabetes. The CPG have provided standards of care for Thai patients with T2D and encouraged using HbA1c as a marker of glycemic control, leading to an increase in the percentage of annual HbA1C testing in T2D from 17% in 2003 to 77.6% in 2014 [19, 20]. Accordingly, Thai patients with T2D have received appropriate medical treatment resulting in alleviating diabetic complications.

Our study indicated the prevalence of DR significantly differed at each hospital level. The DR prevalence in regional hospitals was approximately 12.3% which was the highest compared with provincial and community hospitals. The essential medical facilities and specialists especially in community hospitals may be unavailable; thus, patients with T2D may have limited access to an ophthalmologist [21]. On the other hand, patients with uncontrolled glycemia and T2D with any complication may be referred to higher level hospitals for proper medical management. Consequently, high level hospitals have become permeated with patients with T2D and DR. However, the study did not include patients with T2D visiting university hospitals including tertiary medical centers; thus, DR prevalence may have been underestimated.

Our findings illustrated that the prevalence of DR differed by geographic region. In the central area, DR prevalence was significantly higher than that in other regions. The central area in Thailand consists of the capital and major cities, where appropriate public health services are much more available. Thus, patients can access more services contributing to more reported cases. In addition, the area, which hosts several main agribusinesses, has more than sufficient dietary products combined with improper consumption that might have precipitated vascular complications among patients with T2D [22]. This explanation is also supported by our findings that patients with T2D residing in the central and southern regions tended to present higher BMI when compared with those residing in the northeast.

All Thais have healthcare coverage schemes; we found that DR prevalence among patients with T2D under the SSS was higher than that of patients under other schemes. Basically, the SSS is provided to working age patients who may not follow-up their appointment with medical doctors because the available time conflicts between healthcare service providers and patients [23]. Furthermore, our finding indicated that the proportion of HbA1C level >7% among patients with T2D under SSS was greater than that of patients under other schemes. Thus, the healthcare service access of patients with T2D especially the working age population should be adjusted to create a more appropriate and equitable situation.

The present study found that, a greater duration of DM was associated with DR as a dose response relationship. The finding was consistent with that of a related follow-up study in Spain reporting that 81.1% patients with a diabetes duration of >20 years developed DR [24]. Similarly, a related report in China illustrated that a long duration of DM was attributable to increased DR [25].

We found that DLP was a significant potential risk factor for DR among Thai patients with T2D. Likewise, one recent meta-analysis found that higher LDL cholesterol levels were involved in the progression of DR [26]. Additionally, a Taiwanese cohort study reported that an increase in 1 mg/dl of cholesterol level was associated with increased risk of new-onset DR (hazard ratio 1.01, *p* = 0.001) [27]. The phenomenon can be explained in that the inflammatory process plays a major role in the pathogenesis of DR. In the response to stress especially in DLP, the inflammatory mediators are upregulated leading to abnormal leucocyte-endothelial interactions and eventually retinal microvascular damage [6, 28, 29]. However, a cross-sectional study in southern China reported no association was found between cholesterol levels and DR [30].

Our study revealed that patients with T2D with insulin therapy tended to have a higher risk of DR. Certainly, the insulin was prescribed for patients with T2D with uncontrolled glycemia which were prone to vascular complications. Similarly, several epidemiological studies in China [31], Denmark [32] and Spain [33] supported the fact that insulin therapy is a key factor in DR occurrence. This effect may be explained by two hypotheses. Firstly, the role of osmotic force theory indicates that the rapid decrease in plasma glucose concentration obtained with intensive glucose lowering agents especially insulin therapy lowers the intravascular osmotic pressure, then water retention occurs in the eye vessels which are more sensitive to water [34]. Secondly, the synergistic effect of high dose exogenous insulin and the vascular endothelial growth factor (VEGF) in retinal microvascular endothelial cell trigger vascular proliferation as found in DR [34, 35].

The present study reported that a dose-response relationship existed between HbA1c level and DR prevalence among Thai patients with T2D. Similarly, related studies in the US [36] and China [37] have indicated a significant association between an increase in level of HbA1c and prevalence of DR. Hyperglycemia thoroughly instigates several cascades contributing to retinal vascular endothelial dysfunction, such as oxidative stress, inflammatory processes, protein kinase C (PKC) activation and renin-angiotensin system (RAS). When hyperglycemia proceeds uninhibited, the pathophysiological change will progress with increasing retinal vascular permeability leading to retinal neovascularization [6, 29, 38].

In our study, patients with T2D and elevated PP level more than Q_1_ tended to be at higher risk for DR as a dose-response relationship. One related study in China reported that the presence of DR was 4.6 times that for brachial PP 3^rd^ tertile when compared with that of the 1^st^ tertile [39]. Furthermore, cohort studies in Japan and the UK indicated that PP is a stronger predictor for DR among patients with DM [40, 41]. The phenomenon may be plausibly explained by the hallmark of cardiovascular aging as in arterial stiffness [42]. Arterial stiffness plays a major role in precipitating DR by elevated PP and pulse wave velocity, which are proxy indicators of arterial stiffness [40, 43].

One of the limitations in our study related to the possibility of an underestimated prevalence of DR among T2D because the patients with T2D visiting in the university hospitals in Thailand were not included in this study. Secondly, the classification of diabetic retinopathy was not presented because the limited information of the data which were retrieved from the database Thailand DM/HT. In addition, the study employed serial cross-sectional surveys; thus, illustrating a cause and effect relationship between associated factors and DR would be difficult. Because the data presented in the study were obtained in 2014, 2015 and 2018 in Thailand regarding DM/HT, we are concerned regarding possible missing data from the observational study. However, this represented a large sample size and even though some data might be missing from the nationwide study, the associations between outcomes and factors would still be sufficiently valid and reliable to be presented. The strength of this study was being a large epidemiological study and constituting a nation-wide scope for DR in a Thai T2D population. Thus, the results of the study can be generalized to the whole country and similar populations. Our findings suggested that healthcare services access of patients with T2D should be appropriately provided, and patients with T2D should be regularly assessed for DR. Modifiable risk factors for DR especially HT and DLP should be controlled.

## Conclusion

In conclusion, we reported a decreasing trend in the prevalence of DR among Thai patients with T2D over one half decade. Effective interventions, especially attenuating cholesterol level and controlling HbA1c and blood pressure should be provided to patients with T2D. When these modifiable risk factors are prohibited, DR and other cardiovascular complications such as ischemic heart disease and stroke will be alleviated.
